# Assessing Dietary Quality of Older Chinese People Using the Chinese Diet Balance Index (DBI)

**DOI:** 10.1371/journal.pone.0121618

**Published:** 2015-03-26

**Authors:** Xiaoyue Xu, John Hall, Julie Byles, Zumin Shi

**Affiliations:** 1 Priority Research Centre for Gender, Health and Ageing, School of Medicine and Public Health, Hunter Medical Research Institute, University of Newcastle, Newcastle, Australia; 2 Centre for Clinical Epidemiology and Biostatistics, School of Medicine and Public Health, Hunter Medical Research Institute, University of Newcastle, Newcastle, Australia; 3 School of Medicine, University of Adelaide, Adelaide, Australia; Tufts University, UNITED STATES

## Abstract

**Background/Objectives:**

Few studies have applied the Chinese Diet Balance Index (DBI) in evaluating dietary quality for Chinese people. The present cross-sectional study assessed dietary quality based on DBI for older people, and the associated factors, in four socioeconomically distinct regions in China.

**Methods:**

The China Health and Nutrition Survey (CHNS) involves 2745 older Chinese people, aged 60 or over, from four regions (Northeast, East Coast, Central and West) in 2009. Dietary data were obtained by interviews using 24 hour-recall over three consecutive days. Four indicators: Total Score (TS), Lower Bound Score (LBS), Higher Bound Score (HBS) and Diet Quality Distance (DQD) from DBI were calculated for assessing dietary quality in different aspects.

**Results:**

68.9% of older people had different levels of excessive cereals intake. More than 50% of older people had moderate or severe surplus of oil (64.9%) and salt (58.6%). Intake of vegetables and fruit, milk and soybeans, water, and dietary variety were insufficient, especially for milk and soybeans. 80.8% of people had moderate or severe unbalanced diet consumption. The largest differences of DQD scores have been found for people with different education levels and urbanicity levels. People with higher education levels have lower DQD scores (p<0.001), and people living in medium and low urbanicity areas had 2.8 and 8.9 higher DQD scores than their high urbanicity counterparts (p<0.001). Also, significant differences of DQD scores have been found according to gender, marital status, work status and regions (p<0.001).

**Conclusion:**

DBI can reveal problems of dietary quality for older Chinese people. Rectifying unbalanced diet intake may lead to prevention of non-communicable diseases (NCDs). Dieticians and health care professionals need to increase dissemination and uptake of nutrition education, with interventions targeted at regions of lower socioeconomic status.

## Introduction

Nutritional status is a potentially modifiable factor that impacts quality of life and the prevalence of non-communicable diseases (NCDs), such as cardiovascular disease, type 2 diabetes and cancer [1,2]. Unbalanced consumption of foods high in energy (sugar, starch and/or fat) and low in essential nutrients contribute to energy excess, overweight and obesity, while a healthy and balanced diet is the key to good nutritional status and is necessary for a long and healthy life. Given the link between diet and many NCDs [1,2], there has been growing interest in using indices of dietary quality to evaluate adherence to a healthy and balanced diet in different countries around the world. For instance, the ‘Healthy Eating Index’ was developed to evaluate dietary patterns and diet changes in the US population [3, 4]. In the UK, the ‘Healthy Diet Score’ was developed to investigate factors affecting diet in early old age [5]. The ‘Mediterranean Diet Pattern Score’ has been used to estimate population adherence to the Mediterranean dietary pattern [6, 7], which has been identified as a healthy dietary pattern in southern Italy, Greece, and Spain [8]. In China, two dietary quality indices have been designed: ‘Chinese Diet Quality Index (DQI) ‘[9] and ‘Chinese Dietary Balance Index (DBI)’. Both indices are designed to assess under and over nutrition which are important risk factors in the rise of NCDs among China’s large and rapidly ageing population [10].

The DQI consists of ten components based on the Chinese Dietary Guideline and Chinese Food Pagoda [9]. The DBI was developed from the DQI, to evaluate dietary quality, and is recommended by the Chinese Nutrition Society. The purpose of the DBI is to evaluate and assess the overall dietary quality by four different indicators [10], which are Total Score (TS), Lower Bound Score (LBS), Higher Bound Score (HBS) and Diet Quality Distance (DQD).

To date, few studies have used the DBI to evaluate dietary quality for Chinese people [11, 12], especially older people in different Chinese regions. This study aimed to evaluate dietary quality based on the DBI score, and to examine factors which may be associated with adherence to the DBI score by older Chinese people.

## Methods

### Study design

The China Health and Nutrition Survey (CHNS) is an ongoing open cohort survey which has been conducted from 1989, and has been described in detail elsewhere [13–15]. This survey is an international collaborative project between the Carolina Population Centre at the University of North Carolina at Chapel Hill and the National Institute of Nutrition and Food Safety at the Chinese Center for Disease Control and Prevention.

The CHNS uses a multistage random-cluster sampling process to select samples from nine selected provinces across China. The selected provinces vary according to geography, economic development and health indicators and cover all four major economic regions, i.e. Northeast China (Heilongjiang, Liaoning); East Coast (Shandong, Jiangsu); Central China (Hennan, Hubei, Hunan); and West China (Gunagxi, Guizhou) [16]. Within each province, counties were stratified by different income levels (low, middle and high), and a weighted sampling scheme was used to randomly select four counties. The provincial capital and one other lower income city were selected when feasible (other large cities rather than provincial capitals had to be selected in two provinces). Within each county, one county capital town and three villages within the counties and urban/suburban neighborhoods within the cities were subsequently randomly selected. Finally, twenty households were randomly selected from within each village, town or neighborhood. All individuals in each household (N = 15,648) were interviewed in CHNS [15]. In the present study, 2745 participants aged 60 years or over in 2009 that provide dietary data were included.

Survey protocols, instruments, and the process for obtaining informed consent for CHNS were approved by the institutional review committees of the University of North Carolina at Chapel Hill and the National Institute of Nutrition and Food Safety, China Centre for Disease Control and Prevention. All participants have given their written informed consent [15]. The University of Newcastle, Australia has approved use of data in this study (Approval Number: H-2013-0360).

### Dietary data collection

In the CHNS, dietary assessment is based on a combination of data collected at the individual level and a food inventory taken at the household level. Household food consumption was determined by weighing all food consumed by the household over three consecutive randomly selected days. Household food consumption has determined by examining changes in inventory from the beginning to the end of each day, in combination with a weighing and measuring technique. All foods (including edible oils and salt) remaining after the last meal before initiation of the survey were weighed and recorded. All purchases, home production foods were also recorded. Wasted foods were estimated when weighing was not possible. At the end of the survey, all remaining food have been again weighted and recoded. To collect individual dietary data, every household member (aged 12 years or older) was asked to report all food consumed over the previous 24 hours for each of the three days, whether at home or away from home. Using food models and picture aids, trained field interviewers recorded the types and amounts of food consumed at each meal, and the place of consumption of all food items during the previous day. The amount of food in each dish was estimated from the household inventory and the proportion of each dish consumed was reported by each person interviewed [15].

To check data quality, estimates of each individual’s average daily dietary intakes calculated from the household survey and individual dietary intake based on 24 hour recall data were compared. Where there were significant differences between these estimates, the household and individual questions were revisited, and food consumption was questioned further to resolve these discrepancies. Data quality control was also ensured by a high standard of training of the field interviewers, who were trained for at least three days in the collection of dietary data. Dietary data collection of the survey has been described in detail elsewhere [15, 17–20].

The food codes in CHNS correspond with food names in Chinese Food Composition Table were used for food group classification [21, 22]. Total intakes (in grams) for each food group were calculated. Cooking oil and salt intake from household food consumption data was used to supplement the individual dietary data. Individual cooking oil and salt consumption was calculated according to the total amount of oil and salt consumed in the household divided by the number of individuals per household, and was then adjusted for the proportion of the household energy intake by each individual [23]. The alcohol consumption data were calculated from adult survey. Respondents were asked ‘do you drink any kind of alcoholic beverage (beer, wine or liquor)?’, and were asked further questions on drinking frequency, types and quantity consumed (Liang- Chinese ounce, equal to 50 grams) in a week. The average alcohol consumption (beer, wine and liquor) per day were calculated based on the DBI criteria for alcoholic beverages [10]. For beer consumption, one bottle of beer, equated with 640 ml, was used to calculate the scores, as it is the most common bottle size used in China [24].

Water consumption was also calculated from the adult survey. Participants were asked ‘Do you normally drink plain/bottled water?’, and were asked further questions on drinking frequency and number of cups consumed per day (a cup is about 240 millilitres). Total milliliters of water consumed per day were used to calculate the scores.

### Dietary balance index-07

The purpose of the Chinese DBI-07 (revised from the DBI-2002) is to enable assessment of the overall dietary quality in the Chinese population. DBI-07 contains seven components from the ‘Chinese Dietary Guideline’ and the ‘Chinese Food Pagoda’, including: 1) cereals; 2) vegetables and fruits; 3) dairy products, soybean and soybean products; 4) animal food; 5) condiments and alcoholic beverage; 6) dietary variety; and 7) drinking water [10]. A score of 0 for each DBI-07 component demonstrates meeting the recommended intake amounts. The DBI-07 gives negative scores (range -12–0) to evaluate inadequate food intake levels for vegetables and fruits; dairy products, soybean and soybean products; dietary variety and drinking water, which the dietary guidelines recommend that people should consume ‘plenty’ or ‘sufficient’ amounts of these food groups. The DBI-07 gives positive scores (range 0–12) to assess excessive food intake levels for condiments and alcoholic beverages, which the dietary guidelines recommend that people should ‘reduce’ or have a ‘limited’ amount of these food groups. In addition, DBI-07 gives both negative and positive scores for cereals (range -12–12) and animal food (range -12–8), which the dietary guidelines recommend that people should consume an ‘appropriate amount’ of these food groups [10]. Based on the different intake amounts, individuals have different scores for each component, with the largest absolute score for each component being 12 [10].

The dietary variety component of DBI-07 has 12 identified food subgroups. These include: 1) rice and rice products; 2) wheat and wheat products; 3) corn, coarse grains and products, starchy roots and products; 4) dark-coloured vegetables; 5) light-coloured vegetables; 6) fruits; 7) soybean and soybean products; 8) milk and dairy products; 9) red meat and meat products; 10) poultry and games; 11) eggs; 12) fish and shellfish. A score of 0 on this component means the individual has reached or exceeded the lowest recommended intake of all these food groups (a score of 0 is assigned for each food subgroup), while a score of -12 indicates that they did not reach the lowest recommended intake of any food group (a score of -1 is assigned to each food subgroup). The suggested lowest intake is 5g for soybean and products, and 25 g for the other 11 food subgroups based on Chinese DQI [9]. The details of the other DBI-07 components have been described elsewhere [10–12], and a brief description of each component has been provided in the results.

By summing scores for each DBI-07 component, we calculated four indicators of dietary quality [10]. TS is calculated by adding both positive and negative scores. If the total score is negative, it demonstrates that average food intake is insufficient; if the total score is positive, it demonstrates that the average food intake is excessive. However, a total score of 0 may not necessarily mean that people have a balanced food intake, as it is possible that the positive scores may have offset the negative scores. LBS calculates inadequate food intake by adding the absolute values of all negative scores. HBS calculates excessive food intake by adding all positive scores. DQD assess unbalanced food intake by adding the absolute values of both positive and negative scores [10]. The possible range of TS, HBS, LBS and DQD were: -72 to 44, 0 to 32, 0 to 72, and 0 to 84, respectively [10].

For each component and indicator, a score of ‘0’ indicates an excellent dietary intake (no problem); a score of less than 20% of total score means people have a ‘good’ dietary intake (almost no problem); between 20% and 40% of the total score means people have ‘acceptable’ dietary intake (low level); between 40% and 60% of the total score means people have ‘poor’ dietary intake (moderate level); greater than 60% of the total score means people have ‘very poor’ dietary intake (high level) [10].

### Other variables

Height and weight were measured directly, based on a standard protocol recommended by the World Health Organization, by trained health workers [15]. Body Mass Index (BMI) was divided into four categorical levels based on the criteria recommended by Working Group on Obesity in China [25], which are underweight: BMI<18.5 kg m^−2^; normal: BMI:18.5–23.9 kg m^−2^; overweight: BMI 24.0–27.9 kg m^−2^; general obesity: BMI ≥ 28.0 kg m^−2^. Education level was allocated into four categories from the six education categories in the questionnaire: illiteracy; low: primary school; medium: junior middle school and high: high middle school or higher. Marital status was allocated into two categories (married and other) based on five categories in the questionnaire: married; never married; divorced; widowed; separated. Work status was divided into two levels (Yes/No). Urbanicity is defined by a multidimensional 12-component urbanization index, which captures population density, physical, social, cultural and economic environment, which has been explained in previous studies [26, 27]. Tertiles of the urbanization index were used to define low, medium and high urbanicity in the present study.

### Statistical analysis

Statistical/Data Analysis software package STATA/SE 13.1 (STATA, StataCorp, USA) was used for data analysis. Mean and Standard Deviation (SD) were used to evaluate the average DBI score for each component as the data were normally distributed; ANOVAs were used to assess the association between each component of the DBI by predictor factors; Univariate and multivariable linear regression models were used to explore the association between DQD score and age groups, gender, marital status, education levels, work status, BMI, urbanicity levels and the four Chinese regions.

## Results

Of 2745 participants, 57% (n = 1563) were aged 60–69 years and 43% (n = 1182) were aged 70 or above; 47.4% were men and 52.6% were women; approximately half of participants (n = 1341) had normal BMI (18.5–23.9 kg m^−2^). Characteristics of study participants are shown in Table 1.

**Table 1:** Characteristics of study participants.

	Men		Women	
	n	%	n	%
Total	1300	47.4	1445	52.6
***Age group (years)***				
60–69	774	59.5	789	54.6
70 or above	526	40.5	656	45.4
***Marital Status***				
Married	1103	85.6	926	64.7
Other marital status*	186	14.4	506	35.3
***Work Status***				
No	865	66.9	1145	79.6
Yes	428	33.1	293	20.4
***Education level***				
Illiteracy	176	13.7	642	44.8
Low	573	44.5	539	37.6
Medium	282	21.9	140	9.8
High	256	19.9	113	7.9
***BMI***				
Underweight	101	8.2	121	8.8
Normal	669	54.3	672	48.8
Overweight	380	30.8	417	30.3
Obesity	83	6.7	166	12.1
***Urbanicity levels***				
Low	457	35.2	475	32.9
Medium	426	32.8	475	32.9
High	417	32.1	495	34.3
***Regions***				
Northeast	241	18.5	244	16.9
East Coast	321	24.7	352	24.4
Central	404	31.1	458	31.7
West	334	25.7	391	27.1

*Other marital status includes divorced; widowed; separated and never married.

Table 2 shows score for components of food intake, and percentage of older Chinese people with each score (using energy intake of 2000 kcal per day as an example). Excessive cereal intakes were common, with 68.9%of older people having a positive score. In contrast, inadequate intakes of vegetables and fruits, milk and soybeans, drinking water, fish and shrimps, and eggs were common, with the score for most older people being in the negative range. Dietary variety was also below recommended levels, with almost all (99.8%) of the older people being in the negative scores. More than 90% of people had moderate or severe levels of insufficient dairy consumption. In contrast, more than half of older people had moderate or severe surplus of salt (58.6%) and oil (64.9%), and meat was almost as likely to be positive (excessed) as negative (inadequate). Alcohol consumption scores were good for most of the sample, with 90.8% of men and 89.8% of women drinking not at all or not in excess of the recommended amount.

**Table 2:** Score for components of food intake, and percentage of older Chinese people with each score.

	Scores														
Components	Score Range	Subgroups	(-12)-(-11)	(-10)-(-9)	(-8)-(-7)	(-6)–(-5)	(-4)-(-3)	(-2)-(-1)	0	(1)-(2)	(3)-(4)	(5)-(6)	(7)-(8)	(9)-(10)	(11)-(12)
**Cereals**	(-12)-(12)	**Cereals (%)**	0.2	0.4	1.1	2.3	4.7	8.5	14.0	16.1	15.2	12.0	7.2	5.1	13.3
		Intake amount (g)*	<25	25–75	75–125	125–175	175–225	225–275	275–325	325–375	375–425	425–475	475–525	525–575	>575
**Vegetables and fruits**	(-6)-(0)	**Vegetables (%)**				0.5	17.8	46.9	34.8						
		Intake amount (g)*				<1	1–175	175–350	≥350						
	(-6)-(0)	**Fruits (%)**				69.4	16.8	8.8	5.0						
		Intake amount (g)*				<1	1–150	150–300	≥300						
**Dairy, soybean and soybean products**	(-6)-(0)	**Dairy (%)**				92.9	2.4	4.2	0.5						
		Intake amount (g)*				0–100	100–200	200–300	≥300						
	(-6)-(0)	**Soybean (%)**				37.7	4.0	9.1	49.2						
		Intake amount (g)*				<1	1–20	20–40	≥40						
**Animal food**	(-4)-(4)	**Meat (%)**					20.4	10.9	28.1	20.3	20.4				
		Intake amount (g)*					<1	1–25	25–75	75–125	≥125				
	(-4)-(0)	**Fish and shrimp (%)**					72.7	8.5	18.8						
		Intake amount (g)*					<45	<75	>75						
	(-4)-(4)	**Egg (%)**					37.6	18.5	19.6	13.8	10.5				
		Intake amount (g)*					<1	1–25	25–50	50–75	≥75				
**Condiments and alcoholic beverage**	(0)-(4)	**Oil (%)**							35.1	42.1	22.8				
		Intake amount (g)*							≤25	25–50	>50				
	(0)-(4)	**Salt (%)**							41.5	41.1	17.5				
		Intake amount (g)*							≤6	6–12	>12				
	(0)-(4)	**Alcohol (%)**													
		Men							90.8	6.4	2.9				
		Women							89.8	6.9	3.4				
		Intake amount (g)*													
		Men							≤50	50–100	≥100				
		Women							≤30	30–60	≥60				
**Dietary variety**	(-12)-(0)	**Dietary variety (%)**	0.1	4.1	24.6	43.1	23.9	4.0	0.2						
		Intake amount (g)*	For each food subgroup, score ‘0’ for consumption of greater than 25g (soybean is 5g), otherwise score is ‘-1’												
**Drinking water**	(-12)-(0)	**Drinking water (%)**	19.7	10.2	23.1	17.3	13.0	0	16.7						
		Intake amount (g)*	Score ‘0’ for consumption of greater than 1200ml, score ‘-12’ for consumption of less than 100ml. Score decreased 1 with intake amount decrease 100ml from 0 to -12												

* Intake amount use energy intake of 2000kcal as the standard (details of DBI-07 has been described in He et al (2009)[10]).

The distribution of dietary quality for the LBS, HBS and DQD is shown in Table 3. The LBS indicates inadequate food intake levels among older Chinese, with 14.7% of older people having moderate or high prevalence of inadequate food intake. The HBS indicates that 25% of older people have moderate or high level of excessive food intake. DQD score is an indicator to assess the overall imbalance in dietary intake levels, with more than 80% of people having moderate or high level unbalanced food intake. The DQD score was approximately normally distributed (Mean: 36.8; Standard Deviation: 8.59), and the mean DQD tended to be high, suggesting that older Chinese people have a moderate or high level of dietary intake imbalance.

**Table 3:** Distribution of diet quality among older Chinese people (%).

			Distribution of diet quality (%)				
	Indictor	Mean (SD)	No problem	Almost no problem	Low level	Moderate level	High level
**Inadequate Intake**	**LBS**	27.0 (7.1)	0	28.0	57.3	2.5	12.2
**Excessive Intake**	**HBS**	9.7 (5.1)	2.5	29.1	43.4	21.0	4.0
**Overall unbalance**	**DQD**	36.8 (8.6)	0	0.3	18.9	57.3	23.5

• Score range of LBS is 0–72; No problem: 0; Almost no problem:1–14; Low level:15–29; Moderate level:30–43; High level:>43;

• Score range of HBS is 0–32; No problem: 0; Almost no problem: 1–6; Low level: 7–13; Moderate level: 14–19; High level: >19;

• Score range of DQD is 0–84; No problem: 0; Almost no problem: 1–17; Low level: 18–34; Moderate level: 35–50; High level: >50.

Table 4 shows the mean DBI-07 score for LBS, HBS and TS. According to sociodemographic characteristics, we found significant differences between all three dietary quality indicators and age group, education level, BMI, urbanicity levels, and regions (p<0.01). There were no statistically significant differences between LBS and gender, or TS and work status (p>0.05).

**Table 4:** DBI-07 score (Mean and SD) by predictor factors.

Predictor Variables	DBI_LBS	P value	DBI_HBS	P value	DBI_TS	P value
	Mean (SD)		Mean (SD)		Mean (SD)	
**Gender**						
Men	33.4 (8.4)		10.6 (5.1)		-22.8 (10.0)	
Women	33.6 (8.6)	0.44	8.9 (4.9)	<0.001	-24.7 (10.1)	<0.001
**Age groups**						
60–69	33.0 (8.2)		10.5 (5.0)		-22.5 (9.4)	
70 or over	34.3 (8.8)	0.001	8.7 (5.0)	<0.001	-25.6 (10.6)	<0.001
**Marital Status**						
Married	32.9 (8.5)		10.0 (5.1)		-22.9 (9.9)	
Other marital status*	35.5 (8.3)	<0.001	8.9 (5.0)	<0.001	-26.6 (10.0)	<0.001
**Work Status**						
No	32.9 (8.6)		9.0 (4.9)		-23.9 (10.2)	
Yes	35.2 (7.9)	<0.001	11.6 (5.1)	<0.001	-23.6 (9.7)	0.55
***Education level***						
Illiteracy	36.4 (7.9)		9.1 (5.0)		-27.3 (9.9)	
Low	33.9 (8.0)		10.2 (5.1)		-23.6 (9.5)	
Medium	32.1 (8.5)		10.0 (5.0)		-22.0 (9.9)	
High	27.6 (8.0)	<0.001	9.2 (4.8)	<0.001	-18.4 (9.4)	<0.001
***BMI***						
Underweight	36.3 (7.7)		8.6 (5.1)		-27.6 (9.7)	
Normal	34.0 (8.4)		9.9 (5.2)		-24.1 (10.1)	
Overweight	32.0 (8.5)		10.0 (5.1)		-22.0 (9.8)	
Obesity	32.8 (8.4)	<0.001	9.7 (4.6)	0.004	-23.1 (10.0)	<0.001
***Urbanicity levels***						
Low	37.6 (7.3)		11.5 (5.2)		-26.2 (9.6)	
Medium	33.3 (7.6)		9.0 (4.7)		-24.3 (9.6)	
High	29.5 (8.6)	<0.001	8.6 (4.8)	<0.001	-20.9 (10.4)	<0.001
***Regions***						
Northeast	29.9 (8.2)		10.4 (5.6)		-19.5 (9.1)	
East Coast	32.4 (8.4)		9.2 (4.9)		-23.2 (10.3)	
Central	35.6 (8.8)		10.6 (4.9)		-25.0 (10.2)	
West	34.5 (7.5)	<0.001	8.7 (4.9)	<0.001	-25.8 (9.4)	<0.001

*Other marital status includes divorced; widowed; separated and never married.

Associations between participant characteristics and the DQD score are presented in Table 5, and the final multivariable model shows statistically significant differences in DQD according to gender (with men having higher scores than women), marital status (married people had lower scores), BMI (higher scores with increasing weight), work status (higher scores if employed), education levels (lower scores with higher education), urbanicity levels (lower scores in high urbanicity level areas) and regions (higher in central). No significant differences were observed according to age group.

**Table 5:** Linear regression models for DQD, classified by predictor factor.

	DQD	Univariate Model		Final Multivariate Model	
	Mean (SD)	Coef. (95% CI)	P-value	Coef. (95% CI)	P-value
**Gender**					
Women	42.6 (9.7)	Reference		Reference	
Men	44.0 (9.7)	1.45 (0.73; 2.18)	<0.001	2.40 (1.68; 3.09)	<0.001
**Age groups**					
60–69	43.5 (9.8)	Reference			
70 or over	42.9 (9.6)	-0.60 (-1.33; 0.14)	0.11		
**Marital Status**					
Married	42.9 (9.8)	Reference		Reference	
Other marital status*	44.4 (9.5)	1.51 (0.67; 2.35)	<0.001	1.20 (0.42; 2.00)	<0.001
**Work Status**					
No	41.9 (9.6)	Reference		Reference	
Yes	46.8 (9.2)	4.89 (4.08; 5.70)	<0.001	1.09 (0.28; 1.89)	0.008
***Education level***					
High	36.8 (9.2)	Reference		Reference	
Illiteracy	45.5 (8.8)	8.73 (7.58; 9.88)	<0.001	5.22 (4.00; 6.44)	<0.001
Low	44.1 (9.6)	7.31 (6.21; 8.41)	<0.001	4.15 (3.07; 5.22)	<0.001
Medium	42.1 (9.8)	5.30 (3.99; 6.60)	<0.001	3.24 (2.04; 4.45)	<0.001
***BMI***					
Obesity	42.5 (9.2)	Reference		Reference	
Underweight	44.9 (8.8)	2.43 (0.67; 4.18)	0.007	-0.99 (-2.53; 0.54)	0.20
Normal	43.9 (9.7)	1.38 (0.07; 2.69)	0.04	-0.83 (-1.97; 0.30)	0.15
Overweight	42.0 (10.1)	-0.49 (-1.87; 0.89)	0.49	-1.30 (-2.48; -0.11)	0.03
***Urbanicity levels***					
High	38.2 (9.3)	Reference		Reference	
Low	49.1 (8.3)	10.9 (10.1; 11.7)	<0.001	8.93 (8.02; 9.84)	<0.001
Medium	42.4 (8.3)	4.21 (3.42; 5.01)	<0.001	2.76 (1.92; 3.59)	<0.001
***Regions***					
Northeast	40.2 (10.7)	Reference		Reference	
East Coast	41.7 (9.1)	1.41 (0.30; 2.52)	0.01	0.99 (-0.01; 1.98)	0.05
Central	46.2 (9.9)	5.96 (4.91; 7.02)	<0.001	4.35 (3.39; 5.31)	<0.001
West	43.2 (8.4)	2.97 (1.88; 4.06)	<0.001	0.32 (-0.70; 1.33)	0.54

*Other marital status includes divorced; widowed; separated and never married.

The largest differences in scores have been found between people with different education and urbanicity levels. Illiterate people had the highest score, which was 5.2 times higher than their highest level education counterparts (p<0.001, 95% CI: 4.00; 6.44). The difference of scores between low urbanicity level and high urbanicity was 8.93 (p<0.001, 95% CI: 8.02; 9.84).

## Discussion

This cross-sectional study evaluates the dietary quality of the older population enrolled in CHNS in 2009. DBI-07 can reveal problems of dietary quality for older Chinese people, with LBS and HBS from the DBI-07 reflecting both deficit and surplus of food intake, and DQD assessing the overall imbalance in dietary intake levels. We found that older Chinese people have moderate and high levels of unbalanced diets, and inadequate food intake of vegetables, fruits and dairy is common. Significant differences were found between DQD score by gender, marital status, BMI, work status, education levels, urbanicity levels and regions.

Our results indicate that the diet of Chinese older people largely consists of cereals, with the imbalance being greater in low urbanicity areas. In China, cereals are the major staple food, with traditionally coarse grains and rice in the South, and wheat and wheat products in the North [28]. From our results, it appears that most people (68.9%) have excessive cereal intake, with 25.6% of older people having extreme surplus for cereals (score 7 and above, Table 2). Our study finds that excessive cereal consumption remains a common problem, despite the nutrition transition in China and reduction in cereal consumption since 1982 [28]. Large numbers of people also have moderate or high surplus of oil (64.9%) and salt (58.6%), which may contribute to the increasing prevalence of NCDs or chronic conditions [29]. Studies have shown that high salt intake is associated with stoke and hypertension [29, 30]. A high-fat diet, particularly the overconsumption of cooking oil may be a significant risk factor for obesity in Chinese population [31, 32]. Thus, reducing salt and oil intake will result in a major improvement in public health [29, 31].

Our results show many people have moderate and severe deficits in consumption of vegetables, fruit, milk and soybeans. According to a World Health Report (2014), low vegetable and fruit intake is among the top ten risk factors contributing to mortality, and is associated with death from ischaemic heart disease, gastrointestinal cancer and stroke [33]. Although vegetable and fruit consumption have been reported as increasing during last decade in China [28], the present study shows that the consumption still tends to be low when compared with recommended daily intake. In addition, various factors may cause Chinese people to have low milk and soybean consumption. The most important reason for this is that China’s per capita supply of dairy products is extremely low, accounting for only 3.5% of the world’s total dairy production [34].

Previous research has shown that dietary quality is affected not only by gender, but also by education, occupation and income level [35]—the conventional indices of socioeconomic status [36]. In our study, we also found that the DQD scores are strongly associated with gender and education levels. Furthermore, we found significant differences between DQD scores and the three levels of urbanicity (p<0.001), with people living in high urbanicity areas having lower scores than people living in low urbanicity areas. This is possibly due to the strong association between urbanicity and income levels [37]. Lack of nutritional knowledge, lack of cooking skills and apathy toward nutrition messages are potential reasons for the unhealthy diet among people with lower socioeconomic status [35]. In addition, although common retirement age among Chinese people is 55 for women and 60 for men [38], we found that 26% participants were still working (Table 1). People undertaking paid work had 1.5 higher DQD score than those not working, which indicates that people who do not work have better balanced diets. Although income and social class inequality [37] may have an impact on the food access and dietary quality discrepancies [35, 39], only limited studies have been done in this field in China which indicates the need for further research.

Although the DBI-07 can be used to evaluate the overall dietary quality for Chinese people, there are very few studies that assess the dietary quality by using this score system [10, 12]. Meng et al.’s 2009 study uses one of the components (LBS) of DBI to evaluate dietary quality among older women in south-west China. Our study found similar results to Meng’s study that inadequate food intake is a common problem in older Chinese people [11]. Results from a study for Chinese adults aged 18–59 [12] showed that men had higher DQD scores than women. Higher education level was also related to a better balanced diet and people living in urban areas had lower DQD scores than people living in rural areas. These results are consistent with our present study.

Dietary quality is a potentially modifiable factor that has been associated with NCDs [2, 40]. NCDs such as cardiovascular diseases are to a large extent due to unbalanced diets and physical inactivity. A balanced diet can help people achieve good health and avoid long term diseases and illness. However, many older people lack the nutritional knowledge to make food choices that achieve a balanced diet [41, 42]. Older Chinese people are at high risk of nutritional deficiency and NCDs [1]. These results suggest that more targeted actions should be taken by dieticians and health care professionals to increase dissemination and uptake of mass educational messages.

A limitation of the DBI-07 is that it is not designed for older people in particular, who may have specific dietary needs and are at higher risk of developing NCDs [1]. The extra four general recommendations for older people in the Chinese Dietary Guideline are not reflected in the current DBI-07 [43]. These additional four recommendations suggest that older people should choose foods which are soft, easy to digest and absorb; have access to a variety of foods to maintain their quality of life; attach importance to preventing malnutrition and anaemia; and need to undertake regular physical activity to maintain a healthy weight. These four recommendations need to be further developed for inclusion in the DBI for older Chinese people [1].

The significance of this study is that it is the first to apply different indicators from DBI scores to a large sample of older people in four regions in China. The strengths of this study include the large geographically diverse sample and the use of individual, consecutive three-day recall methods in association with household food inventories to improve the accuracy of reporting of dietary data. We used four indicators from DBI-07 to present the dietary quality of our study participants. The examination of food groups over four diverse socioeconomic regions of China has allowed a more detailed, contextual analysis to be undertaken for a large sample of older Chinese people, in two age cohorts. Moreover, use of the urbanicity index helps us to capture details of population density, physical, social, cultural and economic environments, rather than only an absolute threshold of population and/or population density based on urban and rural measurement. The limitation of the study is that CHNS does not include a representative sample from westernmost of China (such as Tibet and Xinjiang region) where people tend to have ethnically distinct diets and cultural background. However, using a multistage, random-cluster method, the CHNS represents all developmental levels in China, and is geographically representative in nine provinces, which are Northeast China (Heilongjiang, Liaoning), East Coast (Shandong, Jiangsu), Central China (Hennan, Hubei, Hunan) and West China (Gunagxi, Guizhou). As such it reflects significant regional dietary diversity of China.

## Conclusion

The current study suggests that unbalanced dietary consumption is a common problem among older Chinese people. Therefore, the prevalence of diet-related NCDs and age-associated nutritional problems are likely to increase. Targeted education programs to promote adherence to recommended dietary guidelines are crucial for preventing increases in NCDs in China. Moreover, development of the DBI for older Chinese people with age-specific information may help and encourage health professionals and researchers to evaluate older people’s dietary quality.
